# Laboratory, Clinical, and Pathohistological Significance of the Outcomes of Patients with Membranous Nephropathy After 10 Year of Follow-Up

**DOI:** 10.3390/life15081221

**Published:** 2025-08-01

**Authors:** Marko Baralić, Selena Gajić, Mihajlo Kostić, Milorad Stojadinović, Kristina Filić, Danka Bjelić, Vidna Karadžić-Ristanović, Ivana Mrđa, Jovana Gavrilović, Danica Ćujić, Aleksandar Sič, Stefan Janković, Ivan Putica, Sanja Stankovic, Dušan Vićentijević, Maja Životić, Sanja Radojević-Škodrić, Jelena Pavlović, Ana Bontić, Aleksandra Kezić

**Affiliations:** 1 Clinic of Nephrology, University Clinical Center of Serbia, Pasterova 2, 11000 Belgrade, Serbia; 2 Faculty of Medicine, University of Belgrade, Dr Subotića Starijeg 8, 11000 Belgrade, Serbia; 3 Institute for the Application of Nuclear Energy (INEP), University of Belgrade, Banatska 31b, 11080 Belgrade, Serbia; 4 Center for Medical Biochemistry, University Clinical Center of Serbia, 11000 Belgrade, Serbia; 5 Faculty of Medical Sciences, University of Kragujevac, 34000 Kragujevac, Serbia; 6 Institute of Pathology “Dr. Đorđe Jovanović”, Faculty of Medicine, University of Belgrade, 11000 Belgrade, Serbia

**Keywords:** membranous nephropathy, proteinuria, chronic kidney disease, hypertension

## Abstract

Membranous nephropathy (MN) is the most prevalent cause of nephrotic syndrome (NS) in adults, and it can be primary (idiopathic) with an unknown cause or secondary due to a variety of conditions (lupus, infections, malignancies, medications, etc.). It progresses to chronic kidney disease (CKD) in up to 60% of patients, and 10 to 30% develop end-stage kidney disease (ESKD). This retrospective study examines the importance of specific factors, including baseline demographic and clinical data, kidney biopsy PH findings, and selected biochemical parameters, influencing MN outcomes after 10 years of follow-up. The cohort included 94 individuals in whom a diagnosis of MN was established by percutaneous biopsy of the left kidney’s lower pole at the University Clinical Center of Serbia (UCCS) between 2008 and 2013. According to the outcomes, patients were divided into three groups: the recovery (Rec) group, with complete remission, including normal serum creatinine (Scr) and proteinuria (Prt), the group with development of chronic kidney disease (CKD), and the group with development of end-stage kidney disease (ESKD). Nephropathologists graded pathohistological (PH) results from I to III based on the observed PH findings. During the follow-up period, 33 patients were in the Rec group, CKD developed in 53 patients, and ESKD developed in 8 patients. Baseline creatinine clearance levels (Ccr), Scr, and uric acid (urate) were found to be significantly associated with the outcomes (*p* < 0.001). The lowest values of baseline Scr and urate were observed in the Rec group. The presence of acute kidney injury (AKI) or CKD at the time of kidney biopsy was associated with the more frequent development of ESKD (*p* = 0.02). Lower Ccr was associated with a higher likelihood of progressing to CKD (B = −0.021, *p* = 0.014), whereas older age independently predicted progression to ESKD (B = 0.02, *p* = 0.032). Based on this study, it was concluded that the most important biochemical and clinical factors that are associated with the outcomes of this disease are the values of Scr, Ccr, and urate and the existence of CKD at the time of kidney biopsy. Unlike most previous studies, the presence of HTN had no statistical significance in the outcome of the disease.

## 1. Introduction

**Figure 1:**
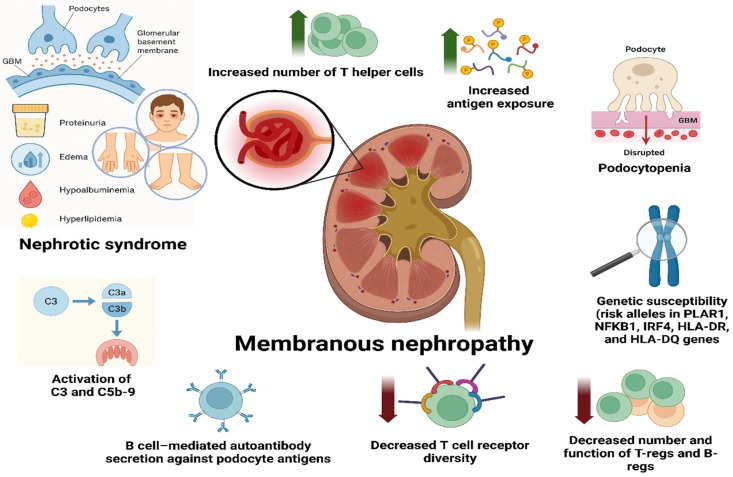
Mechanism of MN and clinical presentation of the disease.

Membranous nephropathy (MN) is one of the most common causes of nephrotic syndrome (NS) in adults, with an incidence of 8 to 10 cases per 1 million people. This disease mainly occurs in men, members of the white ethnic group, and the fifth and sixth decades of life [1]. It can manifest itself as primary MN (PMN) of unknown etiology, which is characterized by the deposition of immune complexes (ICs) on the glomerular basement membrane (GBM). ICs are represented by the podocyte phospholipase A2 receptor (PLA2R) and class G immunoglobulins (IgGs), as well as complement component 3 (C3) [2] (Figure 1). Several studies also discuss genetic predisposition with activation of Wnt1, β-catenin, and renin-angiotensin system (RAS) components in glomeruli [3,4,5]. There is a strong association between MN and nucleotide polymorphism at the HLA-DQA1 locus and the PLA2R1 locus [6] (Figure 1). Additionally, more recent data also indicate the influence of the microbiota on the development of MN [7,8].

**Figure 2:**
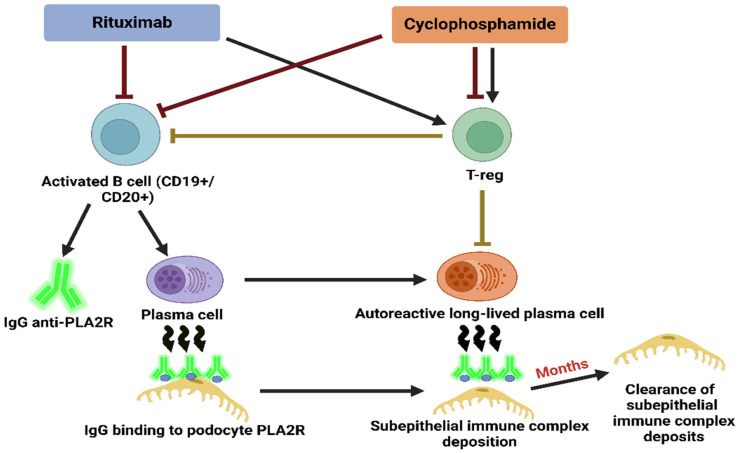
The most common therapeutic modalities for treating MN and the mechanism of therapeutic action.

MN can also be secondary MN (SMN), which is associated with other causes (malignancy, infections, drugs, etc.) and occurs significantly less frequently [1,9,10]. About 80% of patients with PMN develop NS with all clinical and laboratory parameters, and the rest have subnephrotic proteinuria (Prt) [11,12]. Given that one-third of patients can go into spontaneous remission of this disease, without the application of any treatment, after receiving pathohistological (PH) findings, it is usually possible to wait 6 months for the start of treatment, except in forms of the disease that also develop acute kidney injury (AKI) [2,13,14]. However, one-third develop chronic kidney disease (CKD), and the rest develop end-stage kidney disease (ESKD), which is why specific treatment is necessary. Therapy most often involves protocol treatment using corticosteroids and cyclophosphamide, as well as treatment with calcineurin inhibitors and agents that deplete B cells (the monoclonal antibody to the CD-20 molecule) [15,16,17] (Figure 2). With adequate treatment, the possibility of developing ESKD can be significantly reduced by up to 10% [13,16,18].

Given that there is no reliable data on the long-term outcomes of this significant and variable disease in the Republic of Serbia, the aim of this retrospective study is the analysis of the outcomes of patients with PMN who were treated at the University Clinical Center of Serbia (UCCS) during a 10-year follow-up period, as well as the importance of certain factors that most influenced the outcome of the disease that were detected at the time of biopsy (AKI, Prt, hypertension (HTN)) and periodically registered during the disease follow-up period itself (biochemical and clinical parameters). The results were compared with those of patients with SMN.

## 2. Materials and Methods

This study was approved by the Ethical Committee of the University Clinical Center of Serbia (approval number: 890/8, 2018) and conducted following the Declaration of Helsinki and the Ethical Guidelines for Medical and Health Research Involving Human Subjects. Patient informed consent was obtained.

### 2.1. Study Group

This study presents a retrospective analysis of 116 patients with PH findings of PMN and class V lupus nephritis (LN), which, according to its histological characteristics, corresponds to MN, which we classified as SMN. Patients were classified as members of the PMN group after clinical work-up to exclude the presence of malignancy, diabetes, HIV, hepatitis B, and hepatitis C positivity. PH findings were obtained with a percutaneous biopsy of the lower pole of the left kidney in the period from 2008 to 2013. Patients were followed for 10 years. From the total cohort of 116 patients, 22 were excluded from further study because they were continuing their care at other health centers. The final cohort for analysis consisted of 94 patients. All patients were biopsied at the UCCS, and data were used from the IZIS and HELIANT health information systems. Laboratory tests and basic demographic and clinical characteristics, such as edema, systolic and diastolic blood pressure, heart rate (Hr), duration of the disease, presence of HTN, AKI, and CKD, were recorded at the time of kidney biopsy. Every increase in serum creatinine (Scr) values at the beginning of treatment was defined as the presence of AKI or CKD; however, final confirmation of CKD was determined between 3 and 6 months after the start of treatment.

All patients were treated according to the modified Ponticelli protocol. Blood and urine laboratory analyses were performed every month during the six months of induction therapy and then in our outpatient clinic at 3-month intervals in the first 2 years after kidney biopsy. Patients who underwent at least two laboratory analyses from the completed Ponticelli protocol, up to 2 years after kidney biopsy, were eligible for this study. Further biochemical monitoring was performed, depending on the patient, but usually twice a year. After 10 years of follow-up, patients were divided into three groups based on the outcome observed after 10 years of follow-up: Group 1: recovery (Rec), Group 2: CKD, or Group 3: ESKD. We defined recovery as the normalization of Scr and Prt, CKD as an increase in Scr or maintenance of Prt according to KDIGO criteria, and ESKD as a need for kidney replacement treatment.

### 2.2. Laboratory Tests

All biochemical samples were taken early in the morning, 12 h after the patient’s last meal. For biochemical analyses, blood was collected in tubes without anticoagulants. All patient blood was sent to the central laboratory of the UCCS without storage for immediate processing. Biochemical parameters, such as serum urea (Sur), Scr, creatinine clearance (Ccr), uric acid (urate), total proteins (TPs), albumins (Albs), triglycerides (Tgs), total cholesterol (Chol), and Prt, were determined using routine laboratory test procedures on an automated analyzer Architect ci8200 (Abbott Diagnostics, Wiesbaden, Germany). We also subjected the variable A/G, which represents the ratio of serum albumins and globulins, to complete protein analyses. A/G is calculated according to the following formula: TP (g/L)/(TP (g/L) − Alb (g/L)) [19].

Kidney biopsies were performed under ultrasound guidance, with 98% of cases involving sampling of the lower pole of the left kidney. No complications were reported following the procedure. Two tissue cores were obtained from each patient—one for optical microscopy (OM) and one for immunofluorescence (IF) analysis. The sample designated for IF was processed as a frozen section, allowing for the optimal preservation and visualization of immune deposits. IF staining was performed using a standard panel of antibodies: IgA, IgG, IgM, C1q, C3, kappa, lambda, and fibrinogen. In cases of PMN, a characteristic finding was the presence of positive, finely granular staining for IgG and C3 along the GBM, while other immune reactants were typically absent or weak. Biopsy specimens designated for OM were routinely processed and embedded in paraffin. Sections of 4 µm thickness were stained using the following histochemical techniques: Hematoxylin and Eosin (H&E) for general histomorphological assessment of glomeruli, tubules, interstitium, and vessels; Periodic Acid–Schiff (PAS) to evaluate GBM thickening, tubular BM and brush border integrity, and interstitial changes; AFOG (Acid Fuchsin Orange G) to visualize fibrin, immune complexes, proteinaceous material, and collagen deposition; Masson’s Trichrome to highlight collagen deposition and assess the extent of interstitial fibrosis; and Methenamine Silver (Jones stain) to better visualize GBM structure, spikes, and subepithelial deposits.

**Figure 3:**
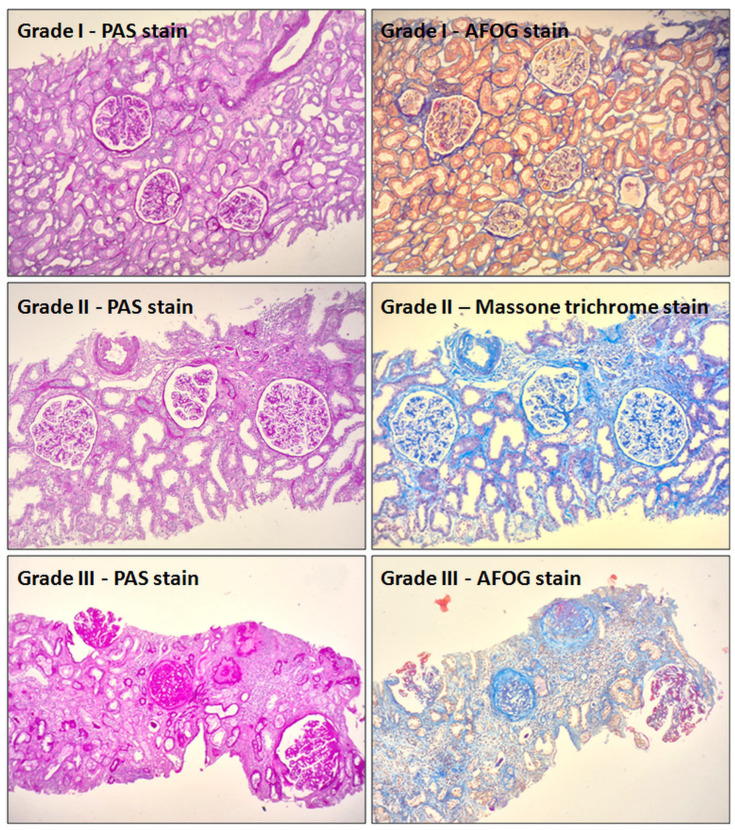
The PH findings included the number of glomeruli, the pattern and type of GBM thickening, the type of deposit on the GBM, and the grade of the disease according to established criteria.

The PH findings included the number of glomeruli, the pattern and type of GBM thickening, the type of deposit on the GBM, and the grade of the disease, according to established criteria [9]. Grading of both interstitial fibrosis and tubular atrophy, as well as chronic glomerular lesions, was defined using the same semi-quantitative scale: Grade I, ≤25% involvement; Grade II, 26–50% involvement; Grade III, >50% involvement. Microscopic evaluation was performed at all magnifications (40×–400×), and the combined information from all five stains (H&E, PAS, AFOG, Masson’s Trichrome, and Jones) was used to ensure diagnostic accuracy and reproducibility [20] (Figure 3).

### 2.3. Statistical Analysis

The normal distribution of continuous variables was tested using the Kolmogorov–Smirnov test. Continuous variables with normal distribution are presented as mean ± standard deviation (SD), and continuous variables that did not show normal distribution are presented as median and interquartile range (IQR). Categorical variables are presented as numbers and percentages. Depending on the data distribution, the differences in measurements were assessed using the paired samples *t*-test or its non-parametric equivalent, the Wilcoxon test. Differences between groups were analyzed using independent samples *t*-tests or Mann–Whitney tests, depending on the distribution. One-factor analysis of variance (ANOVA), i.e., its non-parametric alternative—the Kruskal–Wallis test—was used to analyze the differences between disease outcome groups, depending on the data distribution. The chi-square (χ^2^) test was applied to analyze categorical variables. Multinomial logistic regression was used to analyze the influence of potential predictors on the primary disease outcome. Before conducting the regression analysis, all method assumptions were checked (independence of observations, absence of multicollinearity, and linearity between continuous predictors and logit of the outcome), and extreme values (outliers) were excluded from the final model. Variables that did not meet the assumption of linear association were transformed, and their transformed values were used in the analysis. Statistical analyses were performed using the SPSS statistical package for the social sciences, version 18 (SPSS, Chicago, IL, USA). Statistical significance was defined as *p* < 0.05.

## 3. Results

The investigated demographic, clinical, and laboratory parameters obtained at the time of establishing membranous nephropathy diagnoses are presented in Table 1 and Table 2. In total, 94 patients participated in the study, of which 51 were men (54.3%), and 43 were women (45.7%) (Table 1). The average age was 46 ± 16.40 years (Table 2). The outcome of the disease recorded after ten years of follow-up was as follows: complete remission, i.e., Rec was recorded in 33 patients (35.11%); progression to CKD was found in 53 patients (56.38%); and progression to ESKD was observed in 9 people (9.6%) (Table 1).

The average value of Scr was slightly above the upper limit of the normal range (114.71 ± 89.38 µmol/L) (Table 2). The values of the other investigated laboratory parameters (Prt, TP, Alb, Chol, and Tg) point to overt nephrotic syndrome (Table 2). When a comparison of the baseline variables was performed according to the detected outcomes after 10 years of follow-up, it was found that the highest baseline median value of Scr was in the group that developed ESKD at 210.50 µmol/L [IQR 232], but less in the group that developed CKD at 89 µmol/L [IQR 47] and lowest in the group that achieved Rec at 70 µmol/L [IQR 35], while the value for Ccr was reversed. The highest baseline value of the average Ccr was 122.03 ± 36.55 mL/min and corresponds to the Rec group of patients, with the middle (85.72 ± 39.49 mL/min) in those who developed CKD, while the lowest was in the group that progressed to ESKD. There was a statistically significant difference for Scr and Ccr between the Rec, CKD, and ESKD groups (*p* < 0.001) (Table 3).

The lowest baseline average value of urate was registered in the group of Rec patients (331.64 ± 82.10 µmol/L), which was higher in the group with CKD (400.25 ± 115.79 µmol/L) and highest in the ESKD group (438.86 ± 62.29 µmol/L), and the analyses showed that the difference between the groups was statistically significant (*p* < 0.001). The lowest baseline median value for Prt was in the group of patients who developed CKD, 7.50 g/24 h [IQR 8.90], which was higher in the Rec group, 8 g/24 h [IQR 5.10], and highest in the group of patients who developed ESKD, 8.45 g/24 h [IQR 4.90]. Differences between the groups were not significant (Table 3).

The study also monitored the average values for the baseline TP, alb, and A/G ratio. The lowest average values were in the Rec group, but they were higher in the CKD group and highest in the ESKD group; however, differences between the groups were not significant for any of them (Table 3). Differences between the groups for baseline median Chol and Tg values were also not significant (Table 3).

The type of GBM thickening and Igs deposited were not significantly different between the different outcome groups. According to the type of deposit, the most frequent was the mixed type in 14 patients who recovered, 21 patients with CKD, and 1 with ESKD, while for individual Igs, the most common was IgG in 6 patients who recovered, 17 who developed CKD, and 3 who progressed to ESKD. IgM deposits were slightly less abundant than IgGs, while individual IgAs were rarely seen. There were 8 recoveries, 10 CKDs, and 2 ESKDs without Ig deposition (Table 4).

Differences in PH PMN grades in outcome groups, recorded during the biopsy, were not significant but showed a tendency towards significance (*p* = 0.059). MN PH grade I was recorded in 11 patients (33.3%) in the Rec group, 9 patients (16.98%) in the CKD group, and 1 patient (12.5%) in the ESKD group (Table 4). The MN PH grade III was found in only 24.2% of patients from the Rec group and 26.41% of patients from the CKD group, while in the ESKD group, it was seen in five patients (62.5%) (Table 4).

One of the most important clinical parameters during the first examination of the patient at the time of biopsy is the existence of HTN and AKI or CKD. The presence of HTN at the time of taking the biopsy sample did not significantly affect the outcome of the disease. However, the presence of AKI or CKD at the beginning of the treatment was significantly associated with the outcome (*p* = 0.02) (Table 4). At the beginning of the treatment in the Rec group, 6 (18.2%) patients had AKI; in the CKD group, 17 (30.8%) patients had AKI or CKD; and in the ESKD group, all 8 patients had AKI or CKD (Table 4).

The final multinomial regression model was formed using the following predictors: age, sex, HTN at the time of biopsy, presence of AKI or CKD at the time of biopsy, MN PH grade, serum Alb, Chol, Tg, Prt, Ccr, and the log-transformed urate level. Disease outcomes were classified into three categories: Rec (reference category), CKD, and ESKD. Model evaluation indicated a good fit to the data (χ^2^(164) = 126.69, *p* = 0.986). The model was statistically significant (χ^2^(16) = 32.20, *p* = 0.009), explaining between 29.8% and 36.1% of the variance in the outcome. Overall, it correctly classified 68.1% of cases. Due to a violation of the linearity assumption, the urate level at the time of biopsy was log-transformed. No influential outliers were detected. Ccr and age were statistically significant predictors. Lower Ccr was associated with a higher likelihood of progressing to CKD (B = −0.021, *p* = 0.014), whereas older age independently predicted progression to ESKD (B = 0.02, *p* = 0.032). Log-transformed urate almost reached statistical significance (*p* = 0.057) (Table 5).

## 4. Discussion

MN is the most common cause of NS in adults, with an incidence of 8 to 10 cases per 1 million population. It occurs most often in males in the fifth and sixth decades of life [1]. Our results are consistent with the literature. This disease is immune-mediated and is characterized by thickening of the GBM and subepithelial deposits of Ig and C [9,21,22]. Circulating autoantibodies bind to antigens expressed on the membrane of podocytes with in situ formation of IC, which activate the lectin pathway of the complement with consequent histological podocytopenia and clinically manifested Prt [9,16]. The target antigenic determinants are PLA2R (~70%) and thrombospondin type 1 domain-containing 7A (THSD7A) (2–5%) [2,23,24]. Autoantibodies directed at PLA2R are a significant indicator of PMN [25]. The lack of results from anti-PLA2R antibodies is a study limitation. During the time of kidney biopsy and initiation of treatment for these patients, the Ig titer for PLA2R was not routinely determined at our center. The diagnosis of PMN was based on other investigations and the exclusion of possible causes of SMN, such as infections, malignancies, autoimmune diseases, the use of certain drugs, etc. One of the representatives of SMN is LN class V [2,26]. LN class V was diagnosed in nine patients in our cohort. The determination of anti-PLA2R antibodies at our center has been a standard practice for seven years. However, anti-THSD7A antibodies are still not routinely determined at our center.

MN is characterized by Prt of varying degrees (from subnephrotic to nephrotic), hypoalbuminemia, compensatory hyperlipidemia, clinical edema, and possible HTN [27,28,29,30]. The basic immunosuppressive Ponticelli protocol was initially the first choice for treating MN. Still, other options, such as monoclonal antibodies against the CD20 molecule (rituximab), are often used in therapy, even as a primary choice [13,15,31]. Recently, rituximab has also been a treatment option for these patients at our center; however, at the time of patient inclusion in this study, rituximab was not used routinely.

This disease has different outcomes, from complete or incomplete Rec to the development of CKD and possible consequent ESKD, which develops in up to one-third of patients, according to data in the literature [32,33]. In addition to renal factors and types of immunosuppressive therapy, genetic studies have proved that HLA-DQA1 and PLA2R1 genotype polymorphisms predict responses to immunosuppressive therapy and disease progression [34]. Although the outcomes are known, there are no outcome registries of patients with MN in the Republic of Serbia, which is why this study aimed to track the possible outcomes in a representative cohort during a ten-year follow-up period and to assess how different demographic, clinical, pathohistological, and biochemical parameters affect the course and outcomes of this disease.

Our study showed that during a ten-year follow-up, more than half of the patients developed CKD. Different studies indicate different results [35]. In our study, ESKD developed in 8.51% patients. Similar results were observed in studies conducted in China and the UK [36,37]. Recovery was maintained in 35% of patients. The group of recovered patients characteristically had the highest Ccr. In the study by Kanigicherla and coauthors, a strong association between Scr and Ccr and patient outcomes was found [38]. The value of Ccr at the first presentation of patients with MN is significantly associated with the development of CKD [39]. In our study, lower Ccr at the time of established MN diagnosis was associated with CKD progression. Although recovery was achieved most significantly in the groups with PMN grades I and II, and CKD and ESKD mainly developed in patients with PMN grades II and III, the multinomial regression analysis revealed that the PH grade of MN was not a significant predictor of recovery. The results of this study coincide with the results published by Zhang et al., showing that a higher grade of MN correlates with worse disease outcomes [40]. Additionally, our study indicates that the presence of AKI or CKD at the time of biopsy was significantly more common in the CKD group of patients and patients who will progress to ESKD. However, this was not confirmed in the multinomial regression analysis, which is likely due to an insufficient number of subjects investigated.

Conversely, the obtained results regarding HTN are not in agreement with those of other studies, which suggest the importance of HTN in the progression of kidney disease [18,41]. In our study, HTN had no significant effect on disease outcomes, probably due to adequate antihypertensive therapy, patient compliance, and the presence of pre-existing essential HTN in a significant number of patients before the diagnosis of MN. At the time of kidney biopsy, 64 patients had HTN, among whom 51 had been treated by antihypertensive therapy for a certain period before the established diagnosis of MN. All patients were advised to receive renin–angiotensin–aldosterone system (RAAS) blockade therapy, as well as antilipemic and antiaggregatory therapy.

When looking at the lipid profile, this study found no association between serum Tg and serum total Chol levels with loss of kidney function. However, some authors point out that the Chol level is the main risk factor for the deterioration of kidney function in groups of patients with MN [16,42,43,44]. In addition, in the study by Tran et al. [45], it is stated that lower values of serum Alb represent a negative prognostic factor. Compared to these results, the values of serum Alb, TP, and the A/G ratio in our study did not impact the disease’s outcome. The association between increased urate and the progression of CKD almost reached statistical significance in our study (*p* = 0.057). Previous studies have already confirmed that high concentrations of urate are associated with a higher risk of developing CKD in patients with MN [46,47]. A possible explanation could be the difference in outcome definition and the necessity for a greater number of patients. We found that older age was an independent predictor for progression to ESKD. A similar result was found in the study by Paulo and coauthors [48]. The authors, however, concluded that early diagnosis and prompt treatment of MN will have a positive effect, regardless of patient age. A group of authors from Korea concluded that older age was an independent risk factor for renal outcome risk in patients with MN [49]. It can be concluded that early detection of MN and initiation of therapy are key factors in the treatment of MN, having the most significant impact on outcomes. The degree of advanced fibrosis in the kidneys has a pronounced adverse effect on renal outcome.

### Study Limitations

This study has several significant limitations. First, we did not include the number of relapses in the analysis. Another limitation is that we did not have a group of patients treated with rituximab. That is why we cannot compare the influence of different treatments on the outcome; we can only see the results of one kind of treatment. Finally, the non-detection of the anti-PLA2R antibody raises doubts about the correct and precise inclusion of patients with PMN in the study.

## 5. Conclusions

This study concluded that the level of renal function, particularly the presence of CKD at the time of kidney biopsy, is the most important predictor of this disease’s outcome. PH grade III was the most prevalent finding in the ESKD group, without statistical significance compared with other grades in the outcome groups. HTN, which was confirmed at the time of biopsy, had no significance in the outcome of the disease, in contrast to most studies, which emphasize that HTN is a negative predictor of MN outcomes.

## Figures and Tables

**Table 1:** Basic demographic, clinical, and pathohistological characteristics with recorded clinical outcomes.

Variable	Group	n (%)
Sex	Male	51 (54.3%)
	Female	43 (45.7%)
Outcome	Rec	33 (35.11%)
	CKD	53 (56.38%)
	ESKD	8 (8.51%)
Deposit type Ig	Without Igs	20 (21.3%)
	IgG	26 (27.7%)
	IgM	10 (10.6%)
	IgA	2 (2.1%)
	Mix	36 (38.3%)
HTN before biopsy	No	43 (45.74%)
	Yes	51 (54.26%)
Thickening GBM	Segmentally	16 (17.0%)
	Diffuse	78 (83.0%)

Rec—recovery; Ig—immunoglobulin; CKD—chronic kidney disease; ESKD—end-stage kidney disease; HTN—hypertension; GBM—glomerular basement membrane.

**Table 2:** Clinical and laboratory parameters of patients at the time of kidney biopsy.

Variable	Mean ± SD	Mediana [IQR]
Age, years	45.83 ± 16.40	48.00 [28.0]
sBP, mmHg	139.68 ± 20.70	140.00 [26.0]
dBP, mmHg	83.35 ± 11.50	80.00 [15.0]
Hr, n	80.36 ± 10.90	80.00 [19.0]
Nb Gl OM, n	12.81 ± 7.04	12.00 [11.0]
Prt, g/24 h	9.05 ± 6.23	7.95 [6.5]
Scr, µmol/L	114.71 ± 89.38	86.00 [45.0]
Urate *, µmol/L	378.34 ± 107.05	368.00 [157.0]
TP *, g/L	49.11 ± 9.41	49.00 [15.0]
Alb *, g/L	26.77 ± 7.68	27.00 [11.0]
A/G ratio, n	1.27 ± 0.53	1.21 [0.6]
Chol, mmol/L	7.88 ± 2.56	7.35 [3.5]
Tg, mmol/L	2.86 ± 1.39	2.60 [1.7]
Ccr *, mL/min	95.91 ± 44.36	98.00 [54.5]

sBP—systolic blood pressure; dBP—diastolic blood pressure; Hr—heart rate; Nb Gl OM—number of glomerulus upon optical microscopy; Prt—proteinuria 24 h; Urate—uric acid; Scr—serum creatinine; TP—total protein; Alb—albumin; Chol—cholesterol; Tg—triglyceride; Ccr—creatinine clearance; SD—standard deviation; IQR—interquartile range. * Normally distributed variable.

**Table 3:** Clinical and laboratory baseline parameters of patients according to disease outcomes. Group comparisons were performed using one-way ANOVA or the Kruskal–Wallis test, depending on the data distribution.

Variable	Group	Mean ± SD	Mediana [IQR]	Sig.	Rec	CKD	ESKD
Age, years	Rec	41.21 ± 16.35	44 [31]	0.01	NA		
	CKD	48.54 ± 16.00	48.5 [27]				
	ESKD	55.5 ± 16.79	61 [39]				
sBP, mmHg	Rec	135.0 ± 17.58	135 [28]	NS	NA		
	CKD	141.35 ± 21.38	140 [28]				
	ESKD	147.22 ± 25.63	150 [33]				
dBP, mmHg	Rec	81.58 ± 11.04	80 [18]	NS	NA		
	CKD	83.42 ± 11.83	80 [14]				
	ESKD	89.44 ± 10.14	90 [18]				
Hr, n	Rec	79.64 ± 11.52	80 [18]	NS	NA		
	CKD	79.85 ± 10.25	80 [15]				
	ESKD	86.00 ± 11.79	80 [15]				
Prt, g/24 h	Rec	9.56 ± 6.24	8.00 [5.10]	NS	NA		
	CKD	8.56 ± 6.13	7.50 [8.90]				
	ESKD	10.09 ± 7.29	8.45 [4.90]				
Scr, µmol/L	Rec	77.24 ± 40.78	70 [35]	<0.001	NA	<0.001	<0.001
	CKD	119.29 ± 86.39	89 [47]		<0.001	NA	0.010
	ESKD	240.0 ± 135.03	210.50 [232]		<0.001	0.010	NA
Ccr *, mL/min	Rec	122.03 ± 36.55	116 [34.50]	<0.001	NA	<0.001	<0.001
	CKD	85.72 ± 39.49	80 [50.00]		<0.001	NA	0.078
	ESKD	53.12 ± 47.70	38 [34.80]		<0.001	0.078	NA
Urate *, µmol/L	Rec	331.64 ± 82.10	338 [120]	<0.001	NA	0.009	0.034
	CKD	400.25 ± 115.79	403 [174]		0.009	NA	0.615
	ESKD	438.86 ± 62.29	439 [78]		0.034	0.615	NA
TP *, g/L	Rec	48.03 ± 8.03	48 [12]	NS	NA		
	CKD	49.49 ± 10.57	48 [17]				
	ESKD	51.43 ± 6.19	53 [11]				
Alb *, g/L	Rec	26.03 ± 6.96	27 [10]	NS	NA		
	CKD	26.59 ± 8.26	26 [12]				
	ESKD	31.00 ± 6.0	31.50 [11]				
A/G ratio, n	Rec	1.220 ± 0.39	1.19 [0.49]	NS	NA		
	CKD	1.26 ± 0.58	1.21 [0.63]				
	ESKD	1.58 ±0.69	1.35 [1.18]				
Chol, mmol/L	Rec	8.23 ± 2.53	7.80 [3.60]	NS	NA		
	CKD	7.77 ± 2.61	7.30 [3.40]				
	ESKD	7.08 ± 2.51	6.80 [1.60]				
Tg, mmol/L	Rec	3.16 ± 1.81	2.80 [3.10]	NS	NA		
	CKD	2.72 ± 1.09	2.60 [1.30]				
	ESKD	2.49 ± 1.04	2.45 [1.80]				

Rec—recovery group; CKD—chronic kidney disease; ESKD—end-stage kidney disease; sBP—systolic blood pressure; dBP—diastolic blood pressure; Hr—heart rate; Prt—proteinuria 24 h; Urate—uric acid; Scr—serum creatinine; TP—total protein; Alb—albumin; Chol—cholesterol; Tg—triglyceride; Ccr—creatinine clearance; Sig.—significance; NA—not applicable; NS—non-significant. * Normally distributed data.

**Table 4:** Patient outcomes according to gender, histopathological characteristics of the biopsy specimen, HTN, and azotemia at the time of biopsy.

Variable	Group	Rec	CKD	ESKD	Sig.
Sex	Male	15 (45.5%)	30 (56.6%)	6 (75.0%)	NS
	Female	18 (54.5%)	23 (43.4%)	2 (25.0%)	
Deposit type Ig	Without Ig	8 (24.2%)	10 (18.85%)	2 (25.0%)	NS
	IgG	6 (18.2%)	17 (32.08%)	3 (37.5%)	
	IgM	5 (15.2%)	3 (5.66%)	2 (25.0%)	
	IgA	0 (0.0%)	2 (3.77%)	0 (0.0%)	
	Mix	14 (42.4%)	21 (39.62%)	1 (12.5%)	
Thickening GBM	Segmental	5 (15.2%)	10 (18.85%)	1 (12.5%)	NS
	Diffuse	28 (84.8%)	43 (81.15%)	7 (87.5%)	
MN PH grade	I	11 (33.3%)	9 (16.98%)	1 (12.5%)	NS
	II	10 (30.3%)	25 (47.18%)	2 (25.0%)	
	III	8 (24.2%)	14 (26.41%)	5 (62.5%)	
	LN	4 (12.2%)	5 (9.43%)	0 (0.0%)	
HTN at the time of kidney biopsy	No	14 (42.4%)	15 (28.3%)	1 (12.5%)	NS
	Yes	19 (57.6%)	38 (71.7%)	7 (87.5%)	
AKI or CKD	Yes	6 (18.2%)	17 (32.08%)	8 (100.0%)	0.02
	No	27 (81.8%)	36 (69.2%)	0 (0.0%)	

Rec—recovery; AKI—acute kidney injury; CKD—chronic kidney disease; ESKD—end-stage kidney disease; Ig—immunoglobulins; PH—pathohistology; MN—membranous nephropathy; HTN—hypertension; GBM—glomerular basement membrane; LN—lupus nephritis; Sig.—significance; NS—non-significant.

**Table 5:** Significant predictors comparing outcome groups—multinomial regression.

Predictor	Comparison vs. Recovery	β (SE)	OR	95 % CI for OR	*p*
**Ccr (per 1 mL·min^−1^)**	CKD	–0.021 (0.009)	0.979	0.963–0.996	0.014
**Age (per year)**	ESKD	0.020 (0.009)	1.021	1.002–1.040	0.032

Reference category = recovery; OR—odds ratio (Exp(B)); Ccr—creatinine clearance; CKD—chronic kidney disease; ESKD—end-stage kidney disease.

## Data Availability

The original contributions presented in this study are included in the article; further inquiries can be directed to the corresponding author.

## Abbreviations

The following abbreviations are used in this manuscript.

MN	membranous nephropathy
NS	nephrotic syndrome
PMN	primary membranous nephropathy
SMN	secondary membranous nephropathy
GBM	glomerular basement membrane
Prt	proteinuria
CKD	chronic kidney disease
AKI	acute kidney injury
ESKD	end-stage kidney disease
IC	immune complexes
HTN	hypertension
PH	pathohistological
UCCS	University Clinical Center of Serbia
sBP	systolic blood pressure
dBP	diastolic blood pressure
Hr	heart rate
Gly	glycemic
Sur	serum urea
Scr	serum creatinine
Ccr	creatinine clearance
Tg	triglyceride
Chol	total cholesterol
OM	optical microscopy
IF	immunofluorescence
PAS	Periodic Acid–Schiff
IQR	interquartile range
SD	standard deviation
TP	total protein
Alb	albumins
SEL	systemic lupus erythematosus
KRT	kidney replacement therapy
PLA2R	phospholipase A2 receptor
Ig	immunoglobulin
C	complement components
Rec	recovery
LN	lupus nephritis
Nb Gl	number of glomeruli
AFOG	Acid Fuchsin Orange G
H&E	Hematoxylin and Eosin
